# Augmented Reality-Assisted CT-Guided Puncture: A Phantom Study

**DOI:** 10.1007/s00270-022-03195-y

**Published:** 2022-06-24

**Authors:** Vincent Van den Bosch, Hizirwan Shukri Salim, Njin-Zu Chen, Otto Stroosma, Philipp Bruners, Christiane K. Kuhl, Federico Pedersoli, Peter Isfort

**Affiliations:** 1 Department of Diagnostic and Interventional Radiology, University Hospital RWTH Aachen, Pauwelsstraße 30, 52074 Aachen, Germany; 2 Philips Research Europe, Eindhoven, The Netherlands

**Keywords:** Augmented reality, Phantoms, Imaging, Punctures, Image-guided biopsy

## Abstract

**Purpose:**

To investigate the feasibility of a novel augmented reality system for CT-guided liver interventions and to compare it with free-hand interventions in a phantom setting.

**Methods and materials:**

A newly developed augmented reality interface was used, with projection of CT-imaging in multiplanar reconstruction and live rendering of the needle position, a bull`s eye view of the needle trajectory and a visualization of the distance to the target. Punctures were performed on a custom-made abdominal phantom by three interventional radiologists with different levels of expertise. Time and needle placement accuracy were measured. Two-tailed Wilcoxon signed rank test (*p* < 0.05) was performed to evaluate intraparticipant difference.

**Results:**

Intraparticipant puncture times were significantly shorter for each operator in the augmented reality condition (< 0.001 for the resident, < 0.001 for the junior staff member and 0.027 for the senior staff member). The junior staff member had an improvement in accuracy of 1 mm using augmented reality (*p* 0.026); the other two participants showed no significant improvement regarding accuracy.

**Conclusion:**

In this small series, it appears that the novel augmented reality system may improve the speed of CT-guided punctures in the phantom model compared to the free-hand procedure while maintaining a similar accuracy.

**Supplementary Information:**

The online version contains supplementary material available at 10.1007/s00270-022-03195-y.

## Introduction

Various technologies have been investigated to improve or facilitate CT-guided interventions, like robotic or electromagnetic guidance [1–4]. However, these techniques are often expensive and associated with increased technical complexity and setup time, limiting their usability.

Augmented reality (AR) is a technique which creates an overlay of digital information onto regular vision, typically employing dedicated spectacles. It may be a promising technology for guidance of interventional procedures. However, despite the improvement in the latest devices, the implementation of this technology into clinical practice has remained limited [5, 6].

The aim of this phantom study was to test a newly developed AR-interface featuring the HoloLens 2 headset [7] and to compare AR-assisted with free hand (FH) punctures in simulated CT-guided liver punctures.

## Material and Methods

### Phantom and Tracking System

**Fig. 1:**
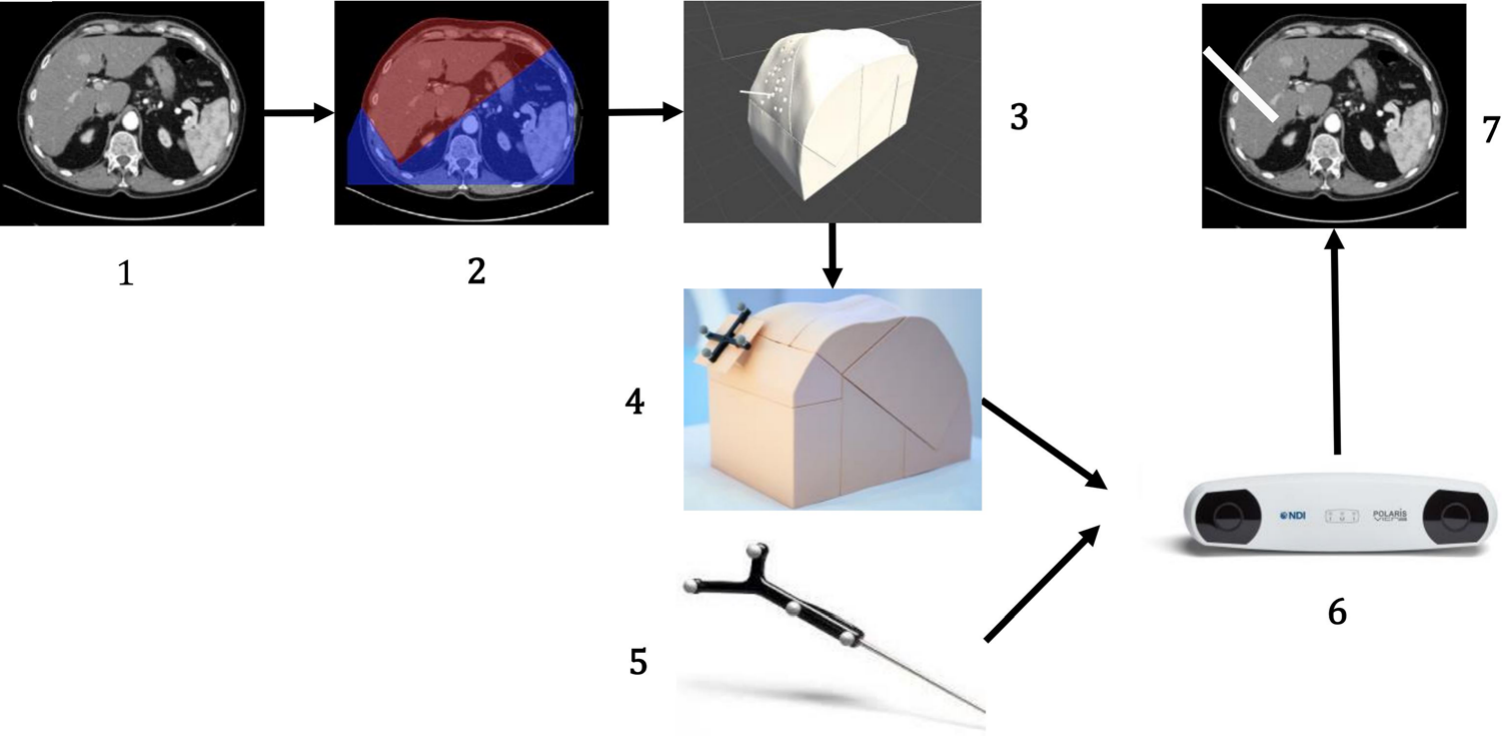
Seven essential steps in creating the CT-simulator: **1** Acquiring a sequence of CT-images. **2** Segmenting the CT-images using 3D-Slicer. **3** Creating the 3D model with marker holder in blender. **4** 3D printing the phantom and placing the marker. **5** Creating the needle with markers. **6** Tracking the phantom and needle with NDI system. **7** Using the tracking information to simulate the needle position over the CT-sequences

**Fig. 2:**
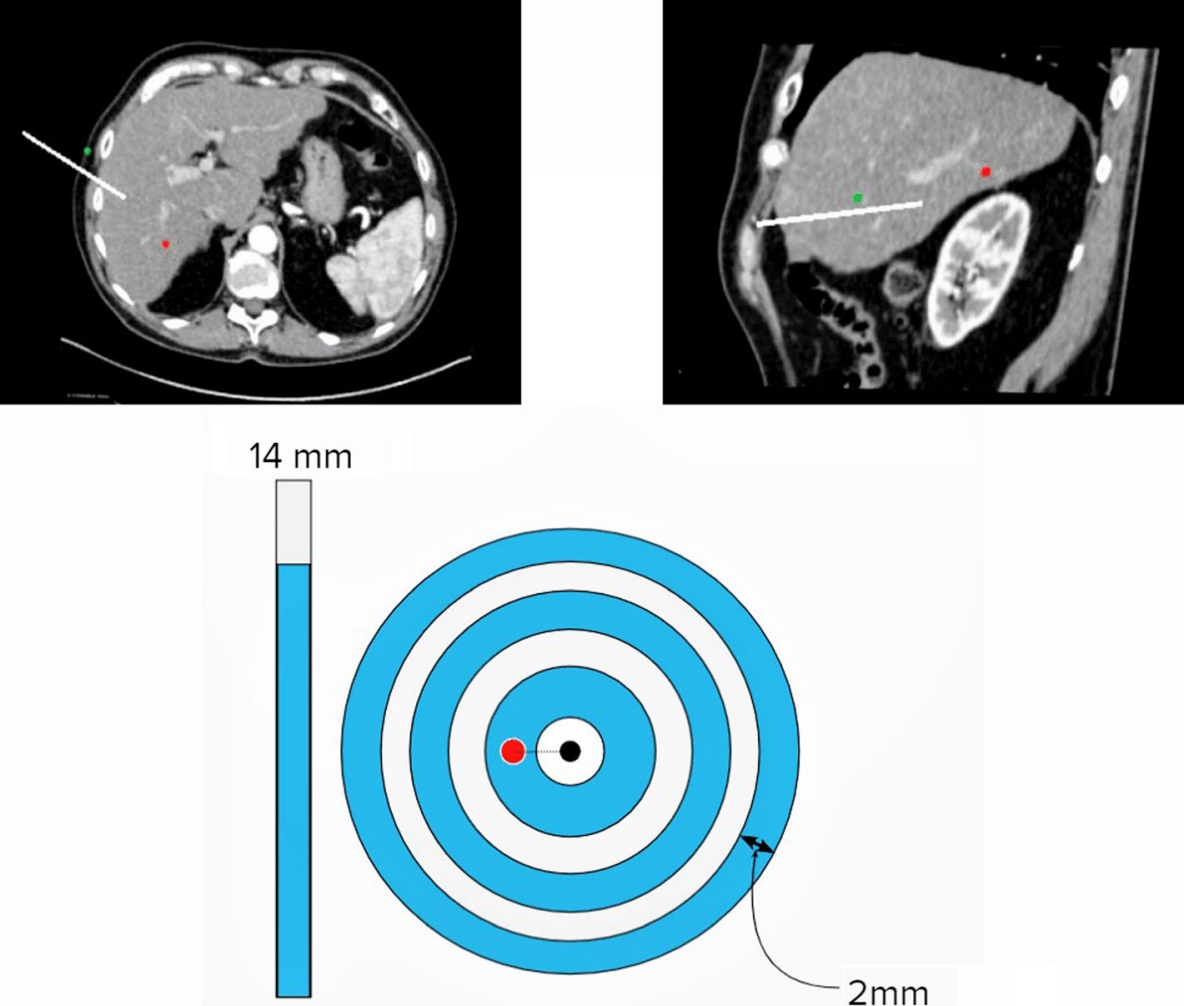
AR-interface as seen by the operator. It consists of an axial CT (top left), a sagittal CT (top right) as well as a bull’s eye with slider. Note the live rendering of the needle and the red dot in both CT-images, being the point-shaped target

The DICOM file of a human abdominal CT was segmented with the application “3D Slicer” [8]. Blender (The Blender Foundation, Amsterdam, Netherlands) was used to refine the phantom model (Fig. 1; step 1 and 2). The model was 3D-printed, with only the liver compartment being filled with tissue-mimicking material (agar–agar). A total of 40 digitalized point-shaped target locations (red dot in Fig. 2) were defined and insertion holes were added to the model (Fig. 1; step 3). All trajectories were out of plane. The puncture needle and the phantom were equipped with optical fiducials (Fig. 1; step 4 and 5) to allow tracking by a FDA approved optical tracking system, NDI Polaris Spectra (Northern Digital, Waterloo, Canada, Fig. 1; step 6). This permitted to track the relative position of the needle to the phantom and superimpose its position on the CT-imaging (Fig. 1; step 7). The superimposed CT-dataset was sent to a virtual environment on the Hololens 2.

### AR Application

This application was developed for the HoloLens 2, with an interface showing CT-imaging in axial and sagittal reformations. Real-time position of the needle was superimposed on the CT-imaging. A visualization with a bull`s eye was integrated, which delivered real-time information of the misalignment between planned and actual needle trajectory. Distance from the needle tip to the target was shown on a slider graph (Fig. 2 and video 1 available online).

### CT-Simulator

The experiments in the FH group were performed with a ‘CT-simulation’ functionality using the phantom. Using the optical tracking, needle projection was visualized within a simulated sequential CT, which was performed upon request of the interventionalist (video 2 available online).

### Operators

Three radiologists without any relevant AR-experience performed the experiments: a resident, a junior staff member and a senior staff member with 0, 2 and 8 years of experience in CT-interventions, respectively. Each operator performed the same number of punctures in FH and AR-condition.

### Procedural Analysis

Time, the interval between the start of the puncture until the operator was satisfied with the needle position, as well as accuracy, defined as distance from the point-shaped targets to the needle tip, were measured. The number of simulated CT-scans that were needed in the FH condition was registered.

### Statistical Analysis

Statistical tests were performed for intraparticipant comparison. A normality test was done using Shapiro–Wilks (*p* < 0.05) method. In case of not normal distributed parameter, a two-tailed Wilcoxon signed rank test was used to evaluate intraparticipant differences. Data analysis was processed using Python 3.9 (Python Software Foundation).

## Results

### Accuracy and Duration

**Fig. 3:**
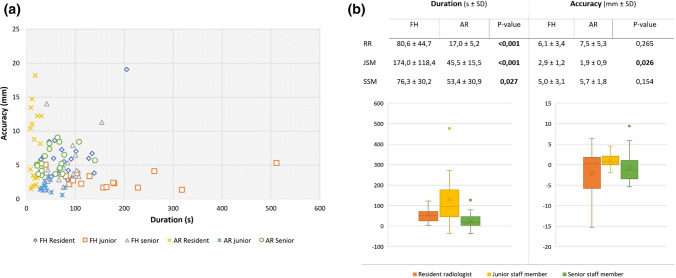
**a**. Graph displaying every single puncture in the experiment with regard to duration and accuracy. **b**. Combination of two box plot graphs and a table describing mean duration and accuracy results for each operator as well as the intraparticipant comparison values. AR: augmented reality; FH: freehand; JSM: junior staff member (yellow); RR: resident radiologist (red); SD: standard deviation; SSM: senior staff member (green)

The resident radiologist performed 40, the junior 30 and the senior 38 punctures (Fig. 3a). All three operators showed a statistically significant improvement in terms of speed when using AR. The mean puncture duration in FH condition was 106.1 ± 82.2 s and 38.8 ± 26.3 s in the AR-condition (Fig. 3b).

Regarding accuracy, the junior staff member was the only operator who significantly improved in the AR-conditions (*p* < 0.05), with a deviation to target of 2.9 ± 1.2 mm in FH conditions and 1.9 ± 0.9 mm in AR-conditions, showing an improvement of 1.0 ± 0.9 mm (Fig. 3b).

A mean of 6.1, 10.3 and 6.0 intermediate CT-scans per trajectory was performed, respectively, by the resident, junior and senior staff member in the FH condition.

## Discussion

In our study, we were able to demonstrate significantly improved procedure times for all levels of experience as well as significantly improved accuracy for the junior staff member.

Scientific evidence on AR-systems for CT-guided procedures is limited. Park et al. [9] published a proof-of-concept study in which they demonstrated that AR-guided punctures decreased the procedure duration of 50%. Li et al. reported on an animal study that compared AR with FH punctures and used a statistical model to compensate for respiratory movement [10]. Long et al. reported on CT-fluoroscopy and AR using both glasses and smartphones and found a comparable needle placement accuracy and reduced procedure time in the AR-condition [11]. Except for the study by Li et al., all the existing studies on the use of AR used the same triple modality 3D abdominal phantom by CIRS. Such phantoms do not regenerate, which can explain the relative low number of punctures performed (less than 20 in each study) [9–12]. The custom-made phantom in this study allowed the liver compartment, composed of agar–agar, to be refilled, and thereby allowing a higher number of punctures. Moreover, the previously published studies used an overlay in which ideal trajectories were overlaid on the physical phantom itself. Contrary to these studies, our setup integrated a phantom which was 3D-printed based on a human abdominal CT. Therefore, we were able to use this CT-dataset for image guidance, creating a more familiar scenario for the radiologists.

This study has several limitations. The number of participants was limited to three operators, which reduced the statistical power of the results. However, the focus of this proof-of-concept study was an intra- rather than an inter-participant comparison. Consequently, the number of punctures was considerably higher compared with similar studies in the literature. Moreover, the comparison was deliberately performed between free-hand punctures, the most commonly used method, and optical tracking with Hololens 2, without considering a group with only optical tracking and traditional monitors without Hololens 2. This was done because the focus of the feasibility study was the comparison of AR-guidance with FH punctures. In a further study, we intend to perform a larger study comparing punctures with optical guidance with and without AR- and FH punctures.

Another limitation is represented by the nature of this is a phantom study, which does not resemble the complexity of live human organs and confounding factors like breathing motion of the patient.

While the use of a CT-simulator might be seen as a limitation, this was deliberately chosen; the CT-simulator eliminated confounders in the creation of the CT and the annotation of needle tip and tumor center position. It may represent an advantage of the study since it eliminates possible confounders and allows unrestricted comparison.

## Conclusion

3D AR-guidance using a headset device may be a promising technique for CT-guided interventions, especially in challenging out-of-plane puncture paths. In the presented feasibility study, it proved to be an effective tool offering a significantly improved procedural time while maintaining a similar accuracy compared to FH punctures. Despite the promising benefits of this phantom study, further developments are necessary before the technique can be adopted into clinical routine.

## Supplementary Information

Below is the link to the electronic supplementary material.

Supplementary file1 (MP4 44000 KB)

Supplementary file2 (MP4 50273 KB)
